# Teachers’ and Learners’ Beliefs About Pronunciation Instruction in Tertiary English as a Foreign Language Education

**DOI:** 10.3389/fpsyg.2021.739842

**Published:** 2021-08-20

**Authors:** Loc Tan Nguyen, Bui Phu Hung, Uyen Thi Thuy Duong, Tu Thanh Le

**Affiliations:** ^1^School of Foreign Languages, University of Economics Ho Chi Minh City, Ho Chi Minh City, Vietnam; ^2^Department of English, Ho Chi Minh City University of Transport, Ho Chi Minh City, Vietnam

**Keywords:** pronunciation instruction, tertiary level, English as a foreign language, teacher beliefs, student beliefs

## Abstract

Recent studies have sought to describe and understand English as a second/foreign language (ESL/EFL) teachers’ pronunciation teaching practices in different contexts, but much less research has examined how teachers and learners perceive pronunciation instruction at tertiary level, especially in EFL settings. The qualitative study reported in this paper extends this line of research by investigating the beliefs of teachers and learners with regard to pronunciation instruction in tertiary EFL education in Vietnam. Data were collected from individual semi-structured interviews with six EFL teachers and focus group interviews with 24 students (four students per group) at a Vietnamese university. The study adopted a content-based approach to qualitative data analysis. The findings show that both the teachers and students considered pronunciation instruction an important component in tertiary EFL programs, which deserves explicit and systematic delivery. The findings suggest that both groups of participants believed communicative pronunciation teaching to have the potential to improve learners’ pronunciation and facilitate their general communicative purposes. The study has implications for language curriculum design and L2 pronunciation teaching and learning.

## Introduction

Beliefs are conceptualized as “propositions individuals consider to be true […], which are often tacit, have a strong evaluative and affective component, provide a basis for action, and are resistant to change” (Borg, 2011, p. 370–371). Teachers’ beliefs play an important role in shaping their pedagogical choices in the classroom, and research into teachers’ beliefs helps advance our understandings about their classroom behaviors (Borg, 2015, 2017). Equally importantly, learners’ beliefs can influence both their learning process and outcomes (Ellis, 2008). As Ha and Nguyen (2021) argue, incongruence in the beliefs of teachers and learners may lead to negative effects but congruence can facilitate the process and outcomes of learning. Thus, it is important for teachers to “make their own beliefs about language learning explicit, to find out about their students’ beliefs, to help their students become aware of and to evaluate their own beliefs and to address any mismatch between their own and their students’ belief systems” (Ellis, 2008, p. 24). Recent decades have seen growing research interest in teacher and learner beliefs about language education generally, but teachers’ and learners’ beliefs about pronunciation instruction in tertiary English as a foreign language (EFL) education are relatively underexplored.

Pronunciation is a fundamental component of communicative competence (Derwing and Munro, 2015; Jones, 2018), since it “permeates all spheres of human life […], in which the speaker and the hearer work together to produce and understand each other’s utterances” (Foote and Trofimovich, 2018, p. 85). In second language (L2) learning, learners who have pronunciation problems are less likely to be properly understood in oral communication irrespective of their excellent grammar and vocabulary (Celce-Murcia et al., 2010; Thomson and Derwing, 2014). Moreover, pronunciation enhances learners’ ability to decode spoken English more efficiently (Adams-Goertel, 2013; Seyedabadi et al., 2015), and research has shown that pronunciation instruction improves listening skills (Ahangari et al., 2015; Kissling, 2018). In this respect, good pronunciation provides grounds for L2 learners’ subsequent development of oral skills.

Within the context of Vietnamese EFL education, however, research shows that Vietnamese EFL teachers tend to teach toward exams, which mainly test learners’ language knowledge (e.g., vocabulary and grammar) rather than language skills such as oral communication (Dang et al., 2013; Nguyen et al., 2015; Ha and Murray, 2021). Such an emphasis on linguistic form in teachers’ classroom practices has resulted in many learners struggling with oral communication despite considerable time and effort spent learning English. Pronunciation as part of oral communication skills, therefore, receives limited attention from Vietnamese EFL teachers. Given that Vietnamese learners of English (VLE) face potential pronunciation problems of both segmentals and suprasegmentals (Lane and Brown, 2010; Avery and Ehrlich, 2013) and that over 2 million students are currently studying English at tertiary institutions nationwide, it is necessary to look into teachers’ and students’ stated beliefs about the role of pronunciation teaching in Vietnamese tertiary EFL education.

Such beliefs, according to Macalister and Nation (2020), are valuable for curriculum designers since student *wants* (what and how learners would like to learn), *necessities* (what learners need to know to be successful in language use), and *lacks* (what learners were not taught or did not practice in their previous learning) are all important. As Macalister and Nation (2020) argue, if students’ learning needs are to be met, it is imperative to address all these three important domains in designing a language curriculum. There is, therefore, a strong need for more research to gain more nuanced insights into teachers’ and students’ beliefs about pronunciation teaching in tertiary EFL settings, in which a large number of L2 teachers and learners are operating. The present study is a timely one to address of this dearth of research by investigating teachers’ and students’ beliefs about pronunciation instruction in Vietnamese tertiary EFL education.

## Literature Review

### Pronunciation: Constructs and Definitions of Terms

There have been two key views of pronunciation: a narrow and a broad view. A narrow view considers pronunciation, the production of individual consonants and vowels in the phonological inventory of a language (Brown, 2000). In contrast, a broad view posits that pronunciation involves all aspects of the oral production of segmentals (consonant and vowel sounds) and suprasegmentals including stress, rhythm, and intonation (Setter and Jenkins, 2005; Derwing and Munro, 2015). This broader view on pronunciation entails the notion that intelligibility and/or comprehensibility is an achievable goal of L2 pronunciation instruction rather than accent reduction. Derwing (2018) defines intelligibility as the extent to which an utterance is actually understood by the listener and comprehensibility as how easy an utterance is to understand.

The question, then, is what type of instruction helps learners achieve intelligibility? One response has been a call for a greater instructional focus on suprasegmentals in keeping with research findings that learners who receive such instruction outperform those whose instruction focuses primarily on segmentals (e.g., Gordon et al., 2013). However, there is not much empirical evidence showing suprasegmental instruction to be an optimal way to help learners achieve intelligibility (Levis, 2018). Numerous scholars have concurred that segmental and suprasegmental features are both important for instruction focused on intelligibility and/or comprehensibility, so both deserve to be adequately addressed in classes (Celce-Murcia et al., 2010; Derwing and Munro, 2015).

### Research on Teachers and Learners’ Beliefs About Pronunciation Teaching and Learning

The past few decades have seen a significant comeback of pronunciation research with particular strands focused on the practice of pronunciation teaching as represented in textbooks, teachers’ cognition, and classroom practices (e.g., Derwing et al., 2012; Foote et al., 2016; Couper, 2017; Nguyen and Newton, 2020). From a teaching perspective, teachers hold strongly to a belief that “pronunciation instruction plays a very important or crucial role in the lives of their students across almost all contexts and situations” (Darcy, 2018, p. 16). For instance, the teachers in research of Couper (2017) cited that pronunciation is an integral part of English learning that helps their learners achieve communicative success. This finding aligns with previous research, which showed that teachers attached remarkable importance to pronunciation in ESL learning (Zielinski and Yates, 2014). Interestingly, the teacher participants in Nguyen (2019) study reported pronunciation to be the most important of all language skills.

Pronunciation research has extended to examine learners’ pronunciation instructional needs. For instance, research by Derwing and Rossiter (2002) involved 100 adult ESL learners in Canada who responded to statements and questions about their pronunciation difficulties and strategies to fix communication breakdowns. The findings showed that over 50% of the students considered pronunciation problems, mainly of segmentals, the main cause of their communication breakdowns. To fix such breakdowns, more than half of the participants reported using paraphrasing, followed by self-repetition (28%), and writing/spelling strategy (7%). The study further showed that most students (90%) were willing to take pronunciation courses. More recently, Nguyen (2019) study showed that the students considered pronunciation to be important in English learning and that VLE face pronunciation problems of both segmentals (i.e., long/short vowels, consonants not existing in Vietnamese, and final sounds) and suprasegmentals (i.e., linking, sentence stress, and intonation). The research also revealed the students’ strong desire for explicit pronunciation instruction. Other studies have also found that students considered pronunciation to be an important feature in English learning (Kang, 2010; Simon and Taverniers, 2011; Levis, 2015; Pardede, 2018). Overall, these studies suggest that students well acknowledged their pronunciation problems and demonstrated considerable interest in learning pronunciation.

Taken together, the studies reviewed above show that both teachers and students are well aware of the important role pronunciation plays in L2 learning. However, research investigating teachers’ and students’ stated beliefs about pronunciation instruction at tertiary level is limited. According to Dalton-Puffer et al. (1997), pronunciation teaching and learning is likely more important in tertiary settings. Nevertheless, Levis (2005) holds that the importance of pronunciation teaching in L2 classes has often been intuitive rather than research-based. The present study extends this line of research by investigating EFL teachers’ and students’ stated beliefs about pronunciation instruction in tertiary EFL education in Vietnam. It seeks to answer the following research questions:

1. How do Vietnamese EFL teachers perceive pronunciation instruction at tertiary level?
2. What beliefs do Vietnamese EFL students hold about pronunciation instruction at tertiary level?
3. Is there any (mis)match between the teachers’ and students’ beliefs about pronunciation instruction at tertiary level?

## Materials and Methods

The lack of pronunciation research within the Vietnamese EFL context motivated the authors to conduct a research project on how teacher professional learning (TPL) equipped Vietnamese tertiary EFL teachers to better teach pronunciation. This section presents the research design and methodology the authors employed to carry out the whole research project.

### Research Setting and Participants

The research project was conducted in a non-major English program at a Vietnamese university. Convenience sampling was employed to select case study participants based on their availability and willingness (Creswell, 2012). An invitation email was sent to all 40 Vietnamese EFL teachers at the university; 15 replied and seven volunteered to participate in the study. One teacher later withdrew, leaving a cohort of six teacher participants (one male and five females), aged 29–52. All the teachers held an MA degree in TESOL or Applied Linguistics and had teaching experience ranging from 6 to 23 years.

To ensure their confidentiality, the teachers are given the pseudonyms Quynh, Phuong, Nguyen, Diep, Khoa, and Na in this report. Twelve student groups of four (six groups in each phase of the research project) taught by each teacher were invited for focus group interviews (FGIs) on a voluntary basis. Each student in each group of each phase was assigned S1–S4 as their pseudonyms. The following sections describe research design and outline how the data were collected and analyzed from which four journal articles have been derived.

### Research Design

The study was carried out within two phases the key findings of which have been disseminated in four journal articles. The phase 1 study established that pronunciation was largely absent from both curriculum documents and assessment and that pronunciation teaching took place in classes but only to a limited extent. The first paper (Nguyen and Newton, 2020) reported on the teachers’ practices and beliefs about pronunciation teaching. The findings showed that the teachers lacked initial training and TPL opportunities in pronunciation pedagogy and that their pronunciation teaching was restricted to error correction through recasts and/or prompts. The second paper showed that students across the six FGIs expressed a strong desire for explicit pronunciation instruction focused on genuine communication (Nguyen, 2019). The results of these two preliminary studies provided grounds for the design of the phase 2 study.

Phase 2 of the research began with the six teachers attending a 3-h TPL workshop focused on the communicative framework of Celce-Murcia et al. (2010, p. 45) for teaching English pronunciation, which includes five stages: (1) *Description and analysis*; (2) *Listening discrimination*; (3) *Controlled practice*; (4) *Guided practice*; and (5) *Communicative practice*. This communicative pronunciation teaching (CPT) model was chosen because the teachers reported lacking expertise in how to teach pronunciation communicatively. After the workshop, each teacher planned one pronunciation lesson applying framework of Celce-Murcia et al. (2010) with one teacher planning two. In total, seven CPT lessons were designed: (1) /i:/ and /ɪ/; (2) /u:/ and /ʊ/; (3) sentence stress; (4) intonation; (5) /ʃ/ and /ʒ/; (6) /tʃ/ and /dʒ/; and (7) final sounds and linking. The teachers then shared and discussed their lesson plans (LPs) as a whole group. In the following semester, they implemented these lessons in one of their scheduled classes, with two lessons taught on the same day. In the week following each teaching session, a review meeting was held for the teachers to discuss their lessons. Upon completion of all lessons, the teachers were invited for individual follow-up interviews and four students from each class for FGIs.

The focus of the third paper was on how TPL assisted the teachers to become more efficient pronunciation instructors. The study showed that TPL led to changes in the teachers’ beliefs about pronunciation teaching and that the TPL workshop successfully enhanced the teachers’ pronunciation pedagogical knowledge and refined their pronunciation teaching skills (Nguyen and Newton, 2021). Following upon the third study, the fourth paper (Nguyen and Hung, 2021) examined the teachers’ implementation of CPT and the value of the CPT approach from both teaching and learning perspectives. The study demonstrated that the teachers constructed their CPT lessons with a wide range of classroom tasks moving from giving explicit phonetic explanation to form-focused and then meaning-focused practice. The study further showed that both the teachers and students responded positively to the CPT model applied across the seven lessons they had experienced, though there were potential limitations for this teaching model to be fully realized.

On the basis of the four papers, the current paper reports on how the teachers and students perceive pronunciation instruction at tertiary level in Vietnam. Although the study was conducted in a particular context of EFL education in Asia, the findings, we believe, will make valuable contributions to the current international literature on English as a second language (ESL)/EFL pronunciation teaching and learning.

### Data Collection and Analysis

Data were collected from curriculum and instructional materials, classroom observations, video-recordings and field notes from the TPL workshop, LPs, semi-structured interviews with teachers, and FGIs with students. These various sources allowed for triangulation of the data to obtain in-depth understandings about the state of pronunciation teaching at the university and the participants’ perceptions and experiences. Data collection took place over a period of 6 months.

In phase 1, the representation of pronunciation in the university EFL program were first examined through curriculum documents and instructional materials, which laid the foundations for subsequent classroom observations and interviews. Twelve 45-min lessons were then observed across the six teachers (two lessons each) to obtain a snapshot of if, what, how much, and how they taught pronunciation in their classes. After that, individual stimulated recall and in-depth interviews were conducted with the teachers, which centred on the rationales for their observed pronunciation teaching practices and their general beliefs about pronunciation instruction. Four students taught by each teacher participated in FGIs to reflect on the efficacy of their teachers’ pronunciation teaching and discuss their own pronunciation instructional needs.

In phase 2, an intervention (i.e., the TPL workshop followed by planning and teaching seven CPT lessons as described in *Research Design*), was carried out. All CPT lessons were observed and audio-video recorded. In total, 24 classroom observations were made across the six teachers, producing approximately 32 h of observation data. After finishing all lessons, the teachers were invited for individual follow-up interviews to reflect on their experiences with the TPL workshop and the CPT lessons they taught. In the last section of their interviews, each teacher was asked: (1) if pronunciation needs to be included in tertiary EFL education; (2) how much time is needed; (3) which features should be targeted; and (4) which teaching approach would best facilitate students’ learning. The rationales for the teachers’ responses to each of these issues were also elicited. Four students taught by each teacher were invited for FGIs. For their part, the students were first asked to give individual responses regarding their experience with the CPT lessons. In the second part, they were asked: (1) how important pronunciation instruction is and should it be taught at tertiary level; (2) how much time is needed; and (3) which teaching approach would best facilitate student learning. The students were also encouraged to elaborate on the reasons for their answers to each question.

The study adopted a content-based approach to qualitative data analysis, which involved an iterative, cyclical, and inductive process of identifying and refining themes and categories in the data set (Duff, 2008). Curriculum analysis was done with reference to a set of 10 research-based principles in pronunciation teaching (Beven, 2012), while the teachers’ observed pronunciation teaching practices in phase 1 were coded using the four-category scheme of Foote et al. (2011). Their LPs and subsequent teaching in phase 2 were analyzed with reference to the communicative framework of Celce-Murcia et al. (2010). The interview data underwent an iterative process of qualitative data analysis following the five steps recommended by Nunan and Bailey (2009, p. 416–424). Through transcribing and then reading the transcripts, initial themes and categories emerged, and were refined through an iterative process of re-reading and refining the thematic categories.

The present paper reports on findings from the final part of the data set regarding the participants’ stated beliefs about pronunciation instruction in Vietnamese tertiary EFL education, drawing on individual semi-structured interviews with the teachers and FGIs with the phase 2 students.

## Results

### How Do Vietnamese EFL Teachers Perceive Pronunciation Instruction at Tertiary Level?

Discussing the necessity of pronunciation instruction in tertiary EFL education, all the teachers stated that it should be included and taught explicitly and systematically. They reasoned that since pronunciation was overlooked in secondary classes, teaching pronunciation in tertiary EFL classes makes up for this lack. For example, Nguyen said:

*In my opinion, (…) secondary teachers only focused on grammar and vocabulary to help students pass the secondary school graduation and university entrance exams. In the end, students entered universities with poor listening and speaking skills, especially pronunciation. So, I think this [pronunciation instruction at tertiary level] is very important if we want students to use English for oral communication effectively*.

The teachers added that teaching pronunciation at tertiary level helps improve students’ pronunciation, listening, and speaking skills, which are essential for their future jobs. As Na commented:

*I think it’s very important to include pronunciation in tertiary English programs because it’s necessary for students to develop pronunciation, listening, and speaking skills. They need these for the workplace after graduation. Also, students were not taught and corrected pronunciation at secondary levels, so their pronunciation and listening and speaking skills are very poor. So, if pronunciation is taught at universities, teachers can raise students’ awareness of how important pronunciation is for their listening and speaking skills*.

The above quotes show the teachers’ strong belief in the need to include pronunciation instruction at university level, which is drawn from their observations that many VLE enter university with poor pronunciation and communication skills. They reported that one factor leading to such discouraging outcomes is how English is commonly taught at secondary schools. As the teachers described, pronunciation needs to be appropriately addressed once students start learning English (normally from Grade 6 in the 12-grade national education system) but Vietnamese secondary EFL teachers mainly focus on teaching vocabulary and grammar to help students pass the national graduation and university entrance exams. Thus, it is important to include and properly teach pronunciation at tertiary level if students are to use English more effectively for oral interaction.

On the question of instructional timeframe, all the teachers reported that 1–2 45-min periods per week is sufficient. They believed that tertiary students as adult learners have to be responsible for their own studies by proactively broadening their knowledge based on what has been taught in class rather than heavily relying on this as the only source of knowledge. They cited that self-study is compulsory at tertiary level, so students need to do more self-practice on pronunciation apart from exercises and activities in class, as illustrated in Quynh’s response:

*I think one to two periods per week is enough. They’re adult learners so mature enough to know what they need to do if they want to be good at English. At university level, they must be more conscious of self-study, not only study what teachers teach in class. Also, if we focus too much on pronunciation in class, I’m afraid students may get bored*.

For focus of instruction, the teachers expressed two opposite views: all features (two teachers) vs. only features causing pronunciation problems to VLE (four teachers). The four teachers who held strongly to their belief that only problematic features should be taught reasoned that since self-study is imperative at tertiary level, students could study other pronunciation features at home. They also commented that students need to take in-class instruction as guidance for more independent practice because pronunciation requires a lot of time and effort to improve. For instance, Khoa remarked:

*At university level, students cannot only study what teachers teach in class as they used to do at secondary schools. If they want to be good, then they are obliged to do more self-study and self-practice at home. So, I think it’s enough to focus on features that commonly cause pronunciation problems to Vietnamese learners only. Later on, they can base on what teachers have taught to do more self-study and self-practice*.

In contrast, Nguyen and Diep believed that pronunciation instruction at tertiary level needs to cover all features because pronunciation was ignored by many secondary EFL teachers and not all students in one class face the same pronunciation difficulties. Like other teachers, Nguyen and Diep also stressed the importance of self-study to tertiary students’ achievement and were well aware of VLE’s potential pronunciation problems. However, they were concerned that without teachers providing basic instruction in class, students’ self-practice of other pronunciation features may be less productive. They added that repeated instructional focus may lead to monotony in classroom learning. Diep elaborated:

*As I’ve just said, secondary teachers do not teach pronunciation but grammar and vocabulary. So, teaching all features to make up for this and to avoid repetition (…). I think teaching a variety of features makes a difference from lesson to lesson and so arouses students’ interest. I agree that Vietnamese learners share common pronunciation problems, but not all students in one class have the same problems (…). Of course, at university level self-study is very important but let us suppose students want to practice everything to improve their pronunciation but teachers only give instruction on some particular features, then how can they practice features that have not been taught? So, teaching all features will make every student aware of their own pronunciation problems and with teachers’ basic guidance they can do more self-practice at home*.

With regard to teaching approaches, the teachers all stated that CPT has the potential to facilitate students’ pronunciation learning. This belief emerged from the teachers reflecting on their practice of correcting learners’ pronunciation errors through recasts and/or prompts as reported in the first paper (Nguyen and Newton, 2020) and their recent teaching experience with the CPT model in the third and fourth papers (Nguyen and Hung, 2021; Nguyen and Newton, 2021). According to the teachers, if pronunciation is taught communicatively, students’ pronunciation, listening, and speaking skills can simultaneously improve through various communication tasks. They believed that fluency in spoken English is essential for students’ future job search as many of them expect to work for companies where English is used for oral communication. Thus, helping tertiary students develop their pronunciation, listening, and speaking skills can increase their opportunity for employment after graduation, and CPT is one of the ways for teachers to do so. For example, Phuong explained:

*If we want to help students improve their pronunciation as well as listening and speaking skills, then (…) teaching pronunciation communicatively is the most suitable. Students can practice not only pronunciation but also listening and speaking skills through communication situations. These are very important for students when they start working after graduation. Most students want to work for foreign companies to get high salaries and other benefits. But, to achieve this goal they firstly have to practice these skills during their English learning at university. This is why I suppose we have to teach pronunciation if students’ desire is to be satisfied*.

Three teachers further considered that CPT can positively change many students’ lukewarm attitudes toward pronunciation practice. They reasoned that CPT can make students more aware of the importance of pronunciation in oral communication and so they will pay more attention to learning and practicing pronunciation. Quynh added:

*I’ve noticed that many students make pronunciation errors in speaking but aren’t aware that they need practice to improve [their pronunciation]. They’re completely wrong when assuming that in communication their messages only need to get through and that’s enough. This really is what makes them ignore pronunciation practice. So, I think (…) [by] teaching pronunciation communicatively, we can make students more aware of how pronunciation improves listening and speaking skills. Then, they’ll realize that pronunciation learning is as important as other language skills*.

Overall, the teachers, despite their different perspectives of what to teach, shared some common beliefs about pronunciation instruction in tertiary EFL education including how much time to spend and how to teach pronunciation effectively within this particular context. We will now turn to examine the students’ stated beliefs about pronunciation instruction in Vietnamese tertiary EFL education.

### What Beliefs Do Vietnamese EFL Students Hold About Pronunciation Instruction at Tertiary Level?

When asked to rate the importance of pronunciation instruction at tertiary level based on a five-point rating scale (where 1 = *not important at all* and 5 = *most important of all language skills*), most students across the six groups (16/24) chose “*very important*,” followed by “*important*” (6/24) and “*most important of all language skills*” (2/24). This indicates that the students were well aware of the important role of pronunciation and how valuable it is to teach pronunciation at tertiary level. They elaborated that pronunciation teaching helps: (1) enhance tertiary students’ listening and speaking skills, (2) foster their confidence in speaking English, and (3) facilitate vocabulary learning.

Believing pronunciation to be one of the most important factors contributing to their oral communicative success, all the students (*n* = 24) explained that when their listening and speaking skills improve, they will be more likely to get a job at foreign companies after graduation. Given their expectation to work for foreign companies where English is used for oral communication, they stressed the potential of fluency in listening and speaking skills in their future job search. This finding lends support to a claim of Newton (2018) that pronunciation, with a close relation to speaking, is essential for work and study. S4 from FGI4 said:

*I think it’s very important and needs to be taught officially like other language skills. If teachers teach pronunciation like this semester, we’ll have more chances to improve listening and speaking skills. After graduation, we’ll have more job opportunities at foreign companies because usually they require applicants to be fluent in spoken English. And if we want to be good at listening and speaking skills, then we have to practice pronunciation more because only when we are good at pronunciation can we understand what people say and make ourselves correctly understood*.

Six students said that improvement in pronunciation, listening, and speaking skills increases their confidence in using English for interaction. They acknowledged that fear of being laughed at pronunciation errors in their speech makes them less willing to communicate. Thus, if their pronunciation improves and people can understand what they say, then they will be more confident in speaking English. As S4 from FGI5 noted:

*Although we have been studying English for more than seven years, secondary teachers mainly taught vocabulary and grammar. This is why when we enter university, many of us cannot speak English. I see that many students are scared of speaking English because they are afraid their pronouncing incorrectly makes people laugh at them. So, I think if teachers help us improve pronunciation, then we’ll gradually be more confident in speaking English*.

Three students further mentioned that pronunciation teaching enhances their vocabulary learning. Trofimovich and Gatbonton (2006) maintain that learning a lexical item involves learning both its meaning and phonological regularities. It is, therefore, disadvantageous to learn new words without learning their pronunciations (Cakir, 2012). In our study, the students believed that being good at pronunciation helps them memorize the meanings of new words more easily and such memory can sustain over time. For example, S2 from FG1 said:

*Moreover, it [teaching pronunciation] also helps me a lot in learning vocabulary. If I’m good at pronunciation, it’s easier for me to learn new words by heart and remember them longer*.

In terms of instructional time, 16 students across the six groups said 1–2 45-min periods per week would be appropriate and eight called for as much pronunciation teaching as possible. The students who recommended 1–2 periods per week identified three different reasons: (1) self-practice is important; (2) they are English non-majors; and (3) too much instruction is boring. Those who suggested as much pronunciation instruction as possible elaborated on the nature of crowded classes in Vietnamese tertiary settings. The data are presented in Table 1.

**Table 1:** Students’ reasons for their suggested pronunciation teaching time.

Reasons	FG1 (*n* = 4)	FG2 (*n* = 4)	FG3 (*n* = 4)	FG4 (*n* = 4)	FG5 (*n* = 4)	FG6 (*n* = 4)	Total (*n* = 24)
1. Self-practice is important	2	2	2	3	2	2	13
2. English non-majors	1	2	1	0	0	2	6
3. Too much focus is boring	0	1	1	1	0	1	4
4. Crowded classes	2	1	1	1	3	0	8

n, number of students.

Like the teachers, more than half of the students (*n* = 16) believed that allocating 1–2 45-min periods per week to pronunciation instruction at university level is adequate. Of these, most students (*n* = 13) reported that such a timeframe is sufficient for teachers to provide phonetic instruction and basic practice in class to help learners do further practice. As S4 from FGI3 commented:

*I think teachers only need to spend one or two periods per week teaching basic theory and instructing us to practice in class. Then, students have to do further practice because at university level students are required to self-study rather than only studying what teachers teach in class*.

This quote shows the students’ awareness that self-study is compulsory at tertiary level. Implied in the students’ responses is a concern that fully relying on teachers’ in-class instruction is not satisfactory but self-study plays an important part in their learning progress. For this reason, they believed that if learners wish to better their pronunciation, further practice outside the classroom is necessary. This finding aligns with the teachers’ stated beliefs about the importance of self-study at tertiary level in Vietnam as reported in section “How do Vietnamese EFL Teachers Perceive Pronunciation Instruction at Tertiary Level?”

Six students stated that 1–2 periods per week is satisfactory for them as English non-majors who only need basic phonetic instruction and practice to guide further practice outside the classroom. They believed only English majors need in-depth understandings about pronunciation, as illustrated in the following comment by S1 from FGI6:

*I think, teachers only need to provide basic pronunciation theory [phonetic instruction]. We’re English non-majors so do not need to understand it as deeply as English majors. Then, teachers instruct us to practice in class as a foundation for us to further practice at home*.

Four students said it might not be a good idea if teachers spend more than two periods per week teaching pronunciation. As evident in the students’ comments, a heavy focus on pronunciation can be tedious and stressful for learners. This is consistent with the teachers’ belief that too much pronunciation instruction can be boring. For example, S1 from FGI3 said:

*I think one or two periods per week is enough. Sometimes, too much teaching [of pronunciation] can be boring and students will be stressed out in learning. Sometimes, it can be counter-productive*.

From a different perspective, the eight students who advocated as much time as possible on pronunciation instruction described the crowded nature of tertiary EFL classes in Vietnam. They reasoned that with a normal size of over 50 students per class, the more time allocated to pronunciation teaching, the more likely it is for all learners in class to have an opportunity for practice. They also said that this timeframe enables teachers to correct learners’ pronunciation errors. The following comment by S2 from FGI1 illustrates such an expectation of communication tasks and error correction:

*I do not know how many [periods] is enough but I think the more, the better. If we have more time for pronunciation, we’ll have more chances to practice listening and speaking. Classes are crowded, so if there’s only one or two periods, it’s unlikely that everyone in class has a chance to speak and so teachers cannot help correct pronunciation of each student*.

Despite their different views on the instructional timeframe for pronunciation, the students all believed that the CPT approach their teachers recently used would best facilitate student learning. In view of their recent learning experience, the students reported that CPT: (1) provides more communication practice; (2) arouses students’ interest in classroom learning; and (3) provides basic phonetic instruction. We now discuss each of these points in turn.

First, all the students (*n* = 24) stated that CPT provides learners with more opportunities for communicative practice. For example, S1 from FGI1 said:

*If comparing with my previous teachers, I realize that the way my teacher taught pronunciation in this semester is much more effective. Before, teachers only sometimes corrected errors by having us listen and repeat when we pronounced incorrectly. This semester, we had more chances to practice what my teacher taught right away in communication situations. I saw this very interesting because it can help foster our listening and speaking skills a lot*.

This quote illustrates the students’ favorable attitude toward communication tasks CPT presents. Comparing the way their teachers recently taught pronunciation with their previous learning experience, i.e., teachers correcting pronunciation errors through recast and/or prompts, the students considered CPT a more effective approach. Since the opportunity for communication output is attributed to proficiency development in L2 learning (Ellis, 2005), learners need opportunities to practice the new targeted features in more spontaneous interaction (Nation and Newton, 2009). As the students saw it, CPT provides learners with diverse communicative tasks through which they can develop their listening and speaking skills.

Second, most students (18/24) said that CPT arouses learners’ interest in classroom learning. The students’ responses suggest that the classroom atmosphere has an influence on their interest and motivation in learning and practicing pronunciation. Reflecting on their recent experience, the students believed that CPT creates a more relaxing atmosphere *via* a range of communicative tasks, which makes learners more willingly involved in classroom learning. As S3 from FGI2 clarified:

*I think the way my teacher taught pronunciation this semester is more interesting and helpful than teachers only sometimes correcting errors by asking us to listen and repeat in previous semesters. My teacher provided different activities for oral practice in class, making the classroom atmosphere more exciting and so we enjoyed learning more. This way of teaching allows us to practice pronunciation in communication situations, so I feel that our learning is more meaningful. And once students feel they like learning pronunciation, I’m sure they’ll spend more time on practice*.

Third, many students (14/24) stated that CPT helps learners understand basic pronunciation principles, which provide grounds to guide further practice outside the classroom. Implied in the students’ comments is the idea that without basic instruction delivered in class, it is less likely for learners to be successful in self-practice. S1 from FGI4 added:

*Before, teachers only transcribed words on the board and asked us to listen and repeat. And that’s it. This way of teaching is old-fashioned, boring and not as effective as the communicative teaching my teacher used in this semester. Teaching [pronunciation] communicatively helps us know more about pronunciation although basically. For example, my teacher showed us how a sound is pronounced and how to recognize its spellings. She also taught us sentence stress and intonation. These are the basis for our further practice at home*.

Taken together, the teachers and students both acknowledged that pronunciation instruction is an integral component in EFL programs at tertiary level that merits explicit, systematic delivery. They also believed CPT to be a promising approach, which has the potential to facilitate learners’ pronunciation, listening, and speaking skills within this EFL context.

## Discussion and Implications

The study shows an alignment in the teachers’ and students’ beliefs that pronunciation deserves a more substantial role in tertiary EFL education. The finding that the students believed pronunciation instruction to be an essential component at tertiary level confirms previous research. For example, the study of Kang (2010) with ESL learners in New Zealand and North America found that most students considered pronunciation an integral part in English learning. Research by Simon and Taverniers (2011) and Nguyen (2019) also showed that a majority of the learner participants believed pronunciation to be an important feature in oral communication. This finding strengthens a claim that pronunciation is an important part in ESL/EFL education (Foote et al., 2016; Darcy, 2018; Derwing, 2018; Jones, 2018). Nation and Newton (2009, p. 76) have also asserted that pronunciation deserves special attention in the ESL/EFL classroom to help learners “quickly develop a stable pronunciation, and become familiar with the patterns and rules that work within the second language.” In our study, both the teachers and students believed that pronunciation instruction advances learners’ fluency in listening and speaking skills, which are essential for students’ future work upon graduation. However, they reported that pronunciation was neglected at secondary schools and so one possible way to fill this gap is to teach pronunciation explicitly and systematically at tertiary level. On the basis of the study findings, it may be important that tertiary curriculum designers in Vietnam include pronunciation in their EFL programs in order to address the *necessities* and the *lacks* in the framework of Macalister and Nation (2020). By doing this, they also respond to the call for more pronunciation instruction within the language classroom (Isaacs, 2009; Derwing and Munro, 2014, 2015; Couper, 2017).

The study also demonstrates consistency between the teachers’ and students’ beliefs about pronunciation instruction at tertiary level. While both groups suggested 1–2 45-min periods on a weekly basis to be suitable for pronunciation teaching in tertiary settings, some students believed that the more pronunciation is taught, the more students can improve their pronunciation, listening, and speaking skills. This finding in part aligns with previous studies, in which learners expressed a strong desire for more pronunciation instruction (Derwing and Rossiter, 2002; Foote et al., 2011; Pardede, 2018). Despite their common view on how much to teach, the teachers held different beliefs about what pronunciation features need to be targeted in class. Consistent with the functional load principle in pronunciation teaching (Munro and Derwing, 2006), some teachers reported that pronunciation instruction only needs to cover features that cause common problems to VLE in keeping with their belief in the facilitative role of self-study to student learning. This finding lends support to an argument of Timperley et al. (2008) that self-regulated learning is important for all learners. Research by Little (2007), Schunk and Zimmerman (2008), and Nguyen (2009) has also highlighted the influential role of self-regulated learning in learners’ language development. In contrast, other teachers believed that teaching all features evades monotony in classroom instruction and accommodates different learners’ pronunciation difficulties. Leading scholars in the field have concurred that for L2 learners to achieve intelligibility and/or comprehensibility, pronunciation instruction needs to address diverse features ranging from sounds to prosody (Derwing, 2008, 2018; Celce-Murcia et al., 2010). Thus, it is necessary that instructors teach different pronunciation features with priority slightly shifted to students’ instructional needs (Derwing and Munro, 2015).

Finally, the study shows that the teachers and students articulated clear beliefs about how CPT can facilitate students’ learning. CPT immerses L2 learners in language use through communication tasks, in which they have the opportunity to practice the newly-acquired phonological features and get feedback on their production (Celce-Murcia et al., 2010; Avery and Ehrlich, 2013). Based on their recent teaching and learning experiences, both the teachers and students believed that CPT can help learners simultaneously improve pronunciation, listening, and speaking skills, the fluency of which is essential for learners’ employment where spoken English is required for daily communication. This finding strengthens a claim of Adams-Goertel (2013) that the purpose of teaching pronunciation is for learners to develop oral communication that serves their individual communicative purposes. Since learners may have different beliefs about what is helpful for their learning (Macalister and Nation, 2020), it may be useful that CPT is utilized in the Vietnamese tertiary EFL classroom to expose learners with diverse communication tasks rather than isolated practice of individual pronunciation features. This way, the *wants* in the framework of Macalister and Nation (2020) would be accommodated.

In short, the study has advanced our understandings about teachers and learners’ beliefs about the role of pronunciation instruction in EFL education in a particular tertiary context in Asia. The study findings provide useful insights for curriculum designers within the Vietnamese tertiary EFL context and beyond together with pedagogical implications for L2 pronunciation teaching and learning. These understandings and insights might also be applicable to similar settings such as EFL education in Asia, which involves a considerably large number of L2 teachers and learners.

A possible limitation of the current study is that it only involved a small number of participants from one particular context of EFL education in Vietnam. Therefore, it only provides part of a whole picture of how Vietnamese EFL teachers and learners perceive pronunciation instruction at university level. As such, future research could be conducted at different settings with participation of a larger number of teachers and students so that generalisations can be made to obtain more insights into pronunciation instruction within this particular EFL context.

## Conclusion

This study represents an exploratory step in understanding teachers’ and learners’ beliefs about pronunciation instruction in Vietnamese tertiary EFL education. The findings demonstrate that both the teachers and students considered pronunciation teaching an integral component at tertiary level. More importantly, the study highlights both the teachers’ and learners’ stated beliefs about the benefits of a communicative approach to pronunciation teaching within this EFL context. In light of the study findings, it is important that a place for pronunciation instruction be substantially articulated in EFL programs at Vietnamese universities. A practical first step, we believe, is to include more guidance on pronunciation in course books as they are one of the key sources for teachers to guide their classroom instruction (Derwing et al., 2012; Macalister, 2016). Given the teachers’ and learners’ strong beliefs about the potential of CPT, it might be useful for pronunciation instruction in the Vietnamese tertiary EFL classroom to be delivered communicatively so as to facilitate learners’ communication needs.

## Data Availability Statement

The original contributions presented in the study are included in the article/supplementary material, further inquiries can be directed to the corresponding author.

## Ethics Statement

The study involving human participants were reviewed and approved by Victoria University of Wellington Ethics Committee. The participants provided their written informed consent to participate in this study.

## Author Contributions

All authors listed have made a substantial, direct and intellectual contribution to the work, and approved it for publication.

## Conflict of Interest

The authors declare that the research was conducted in the absence of any commercial or financial relationships that could be construed as a potential conflict of interest.

## Publisher’s Note

All claims expressed in this article are solely those of the authors and do not necessarily represent those of their affiliated organizations, or those of the publisher, the editors and the reviewers. Any product that may be evaluated in this article, or claim that may be made by its manufacturer, is not guaranteed or endorsed by the publisher.
